# Silage maize as a potent candidate for sustainable animal husbandry development—perspectives and strategies for genetic enhancement

**DOI:** 10.3389/fgene.2023.1150132

**Published:** 2023-05-26

**Authors:** Krishna Sai Karnatam, Bikkasani Mythri, Wajhat Un Nisa, Heena Sharma, Tarun Kumar Meena, Prabhat Rana, Yogesh Vikal, M. Gowda, Baldev Singh Dhillon, Surinder Sandhu

**Affiliations:** ^1^ School of Agricultural Biotechnology, Punjab Agricultural University, Ludhiana, Punjab, India; ^2^ Department of Plant Breeding and Genetics, Punjab Agricultural University, Ludhiana, Punjab, India; ^3^ International Maize and Wheat Improvement Center (CIMMYT), Nairobi, Kenya

**Keywords:** biomass, silage, digestibility, brown mid-rib, genome-wide association studies, genomic selection

## Abstract

Maize is recognized as the queen of cereals, with an ability to adapt to diverse agroecologies (from 58^o^N to 55^o^S latitude) and the highest genetic yield potential among cereals. Under contemporary conditions of global climate change, C_4_ maize crops offer resilience and sustainability to ensure food, nutritional security, and farmer livelihood. In the northwestern plains of India, maize is an important alternative to paddy for crop diversification in the wake of depleting water resources, reduced farm diversity, nutrient mining, and environmental pollution due to paddy straw burning. Owing to its quick growth, high biomass, good palatability, and absence of anti-nutritional components, maize is also one of the most nutritious non-legume green fodders. It is a high-energy, low-protein forage commonly used for dairy animals like cows and buffalos, often in combination with a complementary high-protein forage such as alfalfa. Maize is also preferred for silage over other fodders due to its softness, high starch content, and sufficient soluble sugars required for proper ensiling. With a rapid population increase in developing countries like China and India, there is an upsurge in meat consumption and, hence, the requirement for animal feed, which entails high usage of maize. The global maize silage market is projected to grow at a compound annual growth rate of 7.84% from 2021 to 2030. Factors such as increasing demand for sustainable and environment-friendly food sources coupled with rising health awareness are fueling this growth. With the dairy sector growing at about 4%–5% and the increasing shortage faced for fodder, demand for silage maize is expected to increase worldwide. The progress in improved mechanization for the provision of silage maize, reduced labor demand, lack of moisture-related marketing issues as associated with grain maize, early vacancy of farms for next crops, and easy and economical form of feed to sustain household dairy sector make maize silage a profitable venture. However, sustaining the profitability of this enterprise requires the development of hybrids specific for silage production. Little attention has yet been paid to breeding for a plant ideotype for silage with specific consideration of traits such as dry matter yield, nutrient yield, energy in organic matter, genetic architecture of cell wall components determining their digestibility, stalk standability, maturity span, and losses during ensiling. This review explores the available information on the underlying genetic mechanisms and gene/gene families impacting silage yield and quality. The trade-offs between yield and nutritive value in relation to crop duration are also discussed. Based on available genetic information on inheritance and molecular aspects, breeding strategies are proposed to develop maize ideotypes for silage for the development of sustainable animal husbandry.

## Introduction

Maize (*Zea mays* L.) is not only an important staple crop for millions of people but also an important crop and now emerging as a type of high-energy silage crop. Maize specifically bred for silage, referred to as silage maize, has the potential to produce high yields with high energy content and can be consumed by ruminants in large amounts. Silage maize continues to be one of the best supplementation options, especially in dry seasons, because of its high dry matter production capacity per unit area, high green mass yield per hectare, high fermentability during storage, and good acceptance by animals (Restle et al., 2006). The corn silage-producing regions are North America (NA), Europe, Asia-Pacific, Latin America, and Middle East Asian countries, with NA countries dominating, with a corn silage market share of 40.1%. By virtue of their advanced technologies, U.S. corn silage production has been steadily increasing in the last 20 years, with a production of 137.675 million tons from 6.71 million acres in 2020 (Statista, 2022). Europe, the second-leading region in the corn silage market, produces 34.2% of the share. The UK is a prominent country in the European region which has boosted its corn silage growth with increased animal product consumption. The remaining regions together contribute 25.7% of the corn silage market. The current market value for corn silage was US$ 342.4 million in 2022 and is predicted to increase to US$ 677.33 million by 2032. The corn silage market is anticipated to show a compound annual growth rate (CAGR) of 7.1% during the forecast period. The adoption of corn silage is rising because of its high energy content and easy digestibility among ruminants. In several countries, grass silage has been the primary forage for dairy cows during winter. However, the dry matter yield, nutritional value, and ensiling characteristics of grass silage are subject to considerable variation. Additionally, grass silage has a lower potential for dry matter and energy intake, thereby limiting its effectiveness in the diets of high-milk-producing dairy cattle (O’mara et al., 1998). In maize, most of the hybrids in the market are developed with specific objectives to improve grain productivity. However, breeding for silage hybrids requires attention not only to grain yield but also for many quality features, including high dry matter yield (DMY), sufficiently high dry matter content (DMC), high feeding quality for ruminants, and high intake by livestock. Breeding for silage also differs from forage hybrids as, for silage, the grain being the richest source of available carbohydrates in the maize plant is an essential breeding goal. The stover quality depends on the climate and growing systems. For instance, longer photoperiods and cooler temperatures produce a higher mass of dry matter in the whole plant. Therefore, the criteria for silage maize improvement will differ by country and the cropping system. We can outline only the generalities of ideal silage maize hybrids.

Initially, genotypes with high grain yield were believed to also produce high forage yield (Nevens, 1933; Nevens and Dungang, 1942). However, subsequent studies demonstrated that the best yields of silage are not achieved with high grain productivity, as the physiology of silage maize hybrids differs from that of grain maize. Many researchers have suggested that the non-grain portion of the maize plant also presents significant opportunities for improvement in yield and quality. To ensure the highest profit from the crop after cattle conversion, a good silage variety must be bred with specific objectives. Silage maize breeders may need to place greater emphasis on selecting for high whole-plant biomass yield rather than focusing on stalk lodging resistance, grain maturity, barrenness, and high grain yield. Breeders may also prioritize the selection of root lodging and general plant health. DMY, whole-plant digestibility, protein content, and the non-structural carbohydrate content of stover are other important traits to ensure high-quality silage. To maximize the selection progress for forage performance, breeders should simultaneously consider both yield and quality traits of stover and grain, as emphasized by Dhillon et al. (1990a).

Genetic improvement of silage maize requires specific approaches. Few reports in the literature have described the results of actual selection schemes aimed at selecting the material for silage performance traits. This review proposes an ideotype for silage maize and conventional and molecular strategies, given the available scientific information on the relevant aspects. In silage maize, green fodder yield and quality parameters are equally important. We have tried to delineate the traits for improvement in two sections:

1. Silage yield components
2. Silage quality attributes

## Silage yield components

### High whole-plant yield

Whole-plant yield comprises all vegetative and reproductive parts devoid of root tissues. For whole-plant yield, several component traits like plant height, stalk girth, leaf traits, and shoot wet and dry weights are positively correlated (Naharudin et al., 2021). The whole-plant yield of maize is generally depicted as biomass. Biomass is the total quantity or weight of plants in a particular area or volume, which is an essential ingredient for increasing silage volume and calorific value (Wei et al., 2009). The sensor-based phenotyping platform technology described by Montes et al. (2011) has shown great potential for high-throughput, non-destructive, and quantitative biomass determination in maize field trials.

Silage maize hybrids should have good and stable biomass yields and grain contents between 46% and 50%, as per the quantity and quality of starch requirements in the diet (Barrière et al., 1997). Pinter (1986) mentioned that, in a continental climate, the ideal silage maize hybrid requires a proportion of at least 30% grain. Vattikonda and Hunter (1983) compared the performance of hybrids for grain and silage at two sites in Ontario. Based on the low correlation (*r* ^2^ = 0.23 and 0.25), they concluded that separate grain and forage performance trials are required to recommend proper production. Researchers differed in their opinions regarding the optimum harvest index for silage maize. Earlier, Perry and Caldwell (1969) and Bunting (1976) suggested that there is no need for grain portion as assimilates stored in the vegetative parts are available in well-digestible form as in the kernels. However, contemporary studies by other authors (Fisher et al., 1968), put forth the need for a large proportion of kernel. Ma and Dwyer, (2012) emphasized the requirement for a ratio of kernel to above-ground biomass for silage hybrids of up to 50%. These discordant findings may be due to differences in climatic conditions, including light intensity and temperature. In terms of whole-plant yield, Dhillon et al. (1990b) and Barrière and Argillier (1993) found that the general combining ability (GCA) had a greater impact than the specific combining ability (SCA). Additionally, the ratio between the variance of SCA and that of GCA was lower for whole plant yield than for grain yield. Bertoia and Aulicino, (2014) reported moderate heritability for stover and whole plant yield traits (h^2^ = 0.41). Melchinger et al. (1992) studied the diversity in European maize inbreds using RFLP markers. They observed higher genetic distance between Flint × Dent than between Flint × Flint and Dent × Dent crosses. Broad-based heterotic groups in Flint and Dent groups in maize were also reported by Dhillon et al. (1993). The dent endosperm is highly preferred for its better starch digestibility, and the selection of dent corn germplasm is being used in maize breeding streams of Western countries (Laflotte et al., 2016). In India, the Flint grain type was preferred in breeding programs, so it is better adapted. Indian fodder maize composite J1006, a ruling variety in the last three decades, was developed using parents Makka-Safed 1 (Flint local) and deep Dent Tuxpeno Planta Baja C7 (Khera et al., 1990).

Increased biomass production depends on a genetic architecture that increases plant growth and produces augmented plant dry matter. The molecular mechanisms behind the biomass increase include genes involved in photosynthetic pathways, cell architecture, and plant growth-promoting regulators (PGPRs). Genotypes with high photosynthetic efficiency were reported to accumulate more biomass (Pena et al., 2017). PGPRs such as auxin, cytokinins, gibberellins (GAs), and brassinosteroids (BRs) can strongly affect the plant’s physiological and biochemical processes. PGPRs such as GA and BRs respond to external sprays to improve biomass accumulation and photosynthetic efficiency in major crops (Han et al., 2018).

### Plant architectural traits for high dry matter yield

It is important to consider plant architectural traits that are closely related to fresh/dry matter yield for silage quality; these include the number of leaves per plant, the number of leaves above the ear, leaf area, plant height, culm thickness, and ear height at harvesting for silage at the dough stage. Leaf angle and orientation attributes, which account for the suitability of maize plants for high-density plantation (Sandhu and Dhillon, 2021), may contribute to enhanced silage productivity. The DMC of stover comprising all the harvested aerial parts, except the ear (consisting of the stalk, leaves, tassel, ear shank, and ear husks), is an important selection criterion for silage maize. High dry matter content in stover helps the plant reach harvest maturity for silage at an earlier stage of development.

It is desirable to select genotypes having higher numbers of leaves to improve quality by increasing the leaf-to-stem ratio (0.2–0.32) as leaves have better digestibility than stalks (Sah et al., 2022). Leaf area is associated with a photosynthetically active surface, and positive relationships with DM at both silking and physiological maturity as well as with grain yield in maize have been reported (Liu G et al., 2017). Earlier, Chase and Nanda (1967) and later Tollenaar and Daynard (1982) reported that although leaf number is a major component of leaf area, among high-yielding hybrids of the same maturity under the same conditions of temperature and photoperiod, variation in total leaf number is quite limited. Several studies on the effects of photoperiod and temperature on final leaf numbers of maize (Bonhomme et al., 1991; Ellis et al., 1992) demonstrated that tropical cultivars were more sensitive to photoperiod than temperate cultivars (Zhang and Mugo, 2001). Therefore, it is important to understand the effect of spatial variation of leaf numbers on DM and grain yields under a broad range of ecological environments (Liu et al., 2020). Li D. et al. (2016) suggested genetic overlap in leaf number and flowering time. The two components of total leaf number (leaves above the ear [LA] and below the ear [LB]) are controlled by contrasting genetic architectures and tend to be under relatively independent genetic control. Due to shared loci between flowering time and LB, flowering time and leaf number exhibited a moderate level of genetic sharing. Additionally, qLA1-1 is a major-effect locus that specifically affects the number of leaves above the primary ear, and it is located in a teosinte-derived region with significant recombination suppression. Leaf orientation with narrow leaf angle and upright leaves results in high biomass accumulation at high densities due to more light penetration (Song et al., 2016). Duvick and Cassman (1999) showed that a more acute leaf angle reduced shading, thus allowing increased photosynthesis per unit of land area. A narrow leaf angle is a highly heritable trait, and crosses of parents with upright leaves tend to produce progeny with upright leaf orientation and vice-versa (Mason and Zuber, 1976). Reduced tassel size frees carbon for investment in other productive plant parts (Duvick and Cassman, 1999). Stem diameter, an important parameter to determine stalk standability, shows low broad sense heritability (28%), indicating more environmental effects. Significant genetic advances in combination with heritability were observed for plant height. According to He and Zhou (2016), maize plant height and ear height inheritance are strongly affected by the environment and exhibit stable heredity with high phenotypic variation.

Delayed senescence or stay-green (SG), the term used to describe genotypes with delayed leaf senescence as compared to reference genotypes (Thomas and Howarth, 2000), has been used as an important crop breeding strategy for higher grain yield (Gregersen et al., 2013). SG was reported as a quantitative trait governed by complex physiological and metabolic networks including chlorophyll efficiency, nitrogen content, nutrient remobilization, and source–sink balance (Munaiz et al., 2020). These traits are associated with higher water and chlorophyll concentrations in the leaves at stover maturity, high stalk and leaf moisture concentrations, and standard senescence under optimal conditions with higher stability (Thomas and Smart, 1993; Bekavac et al., 1998; Munaiz et al., 2020); hence, these may be favorable traits for grain yield, silage yield and quality, double exploitation (grain for feed and stover for bioenergy), stress resistance, etc. Zhang et al. (2019) mentioned that the selection for higher yields has increased stay-green in modern maize hybrids. Maize hybrids with a long period of operational photosynthesis activity produced 24% more stover than non-stay-green (NSG) hybrids during the grain-filling stage; therefore, they are valuable for double exploitation (Zhang et al., 2012). Chibane et al. (2021) also reported longer photosynthesis periods in SG compared to NSG genotypes. SG of the inbred lines of maize is functional and associated with higher N accumulation of matter and uptake after flowering, but a lower N remobilization rate from stover to kernel. Although the proportion of the N of the kernels derived from remobilization was higher in NSG than in SG genotypes (70% vs. 40%), the higher uptake compensates for the lower remobilization, and the N content of the grain was higher in the SG genotypes. With the net effect of higher stover and grain yield, the authors emphasized the potential of SG for breeding for a double purpose (grain for feed and stover for bioenergy). Stay-green also improves produce quality such as sucrose and protein content in maize (McBee, 1983; Gentinetta et al., 1986). Stay-green genotypes can also take up more silicon from the soil, leading to increased lodging resistance (Luyckx et al., 2017). The associations of maize leaf stay-green traits with improved resistance to disease and reduced leaf senescence at high plant densities (Tollenaar, 1991), tolerance to post-flowering drought, high yield, good quality, and increased resistance to pest and lodging (Duvick, 1984; Pommel et al., 2006; Bekavac et al., 2007) have been documented. The SG lines, with greater moisture levels in the stover, could be more adequate for ensiling and the exploitation of residuals for biogas than the moisture of the NSG lines (Grieder et al., 2011).

Sekhon et al. (2019) employed the SysGen framework approach to ascertain source-sink communication as a crucial mechanism accountable for the stay-green trait. Among the 14 high-confidence genes identified, nine genes were found to play a significant role in sugar transport and/or signaling, in addition to the genes associated with staygreen, such as NAC transcription factor, trehalose-6-phosphate synthase, and two xylan biosynthetic enzyme. Higher genetic correlations (≥ 0.8) and the high coincidence of the allelic effects among senescence and agronomic traits highlight the importance of senescence genes (Yannam et al., 2022).

Dry matter content (DMC) is an important criterion for determining the harvest maturity of silage maize, as it is an independent trait that is not entirely determined by flowering time or grain maturity. This indicates the scope of selection for earliness without sacrificing the yield. Silage hybrids should have suitable stalk strength and good resistance to root lodging, as higher planting density stress in forage maize may increase susceptibility to lodging. The longer maize remains in the field, the more vulnerable it is to lodging, but, as silage is harvested at an early stage in development, this lessens the chances of serious lodging.

### Physiological maturity

**FIGURE 1:**
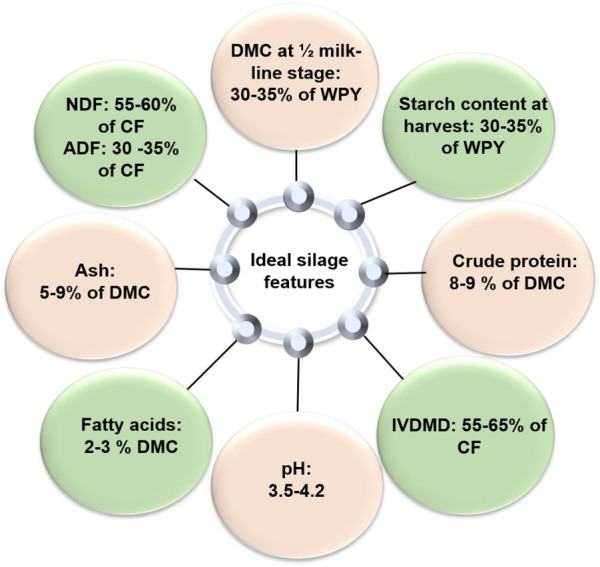
Optimum level of quality parameters for good silage.

Harvesting the crop at the optimum stage for silage is critical. The best physiological maturity stage of maize kernel for silage is the half-milk stage, with optimum moisture of 30%–35%. At the half-milk line stage, half of the kernel is filled with milky endosperm, which highly enhances the fermentation process; the remaining content is hard starch digested by the ruminant (Ma and Dwyer, 2012). Low DMC due to early harvest results in low dry matter silage, which may be bulky and, thus, reduces feed intake and animal performance. A low DMC also results in nutrient losses during ensiling (leachates). A DMC of <30% also increases the risk of bacterial and fungal spoilage. Delay in harvesting also results in low palatability and, hence, reduced DM intake. The advancing maturity of the maize crop during the grain-filling period increases the content of DM and starch and decreases the content of the neutral detergent fiber (NDF) (Phipps et al., 2000). With an increase in starch content, the ADF and NDF contents were found to be decreased due to a negative correlation between them (Sutton et al., 2000). In addition, the vitreousness of kernels (i.e., the proportion of vitreous in the total endosperm) increases with maturity (Correa et al., 2002) and is associated with reduced rumen degradability of starch (Ettle et al., 2001) and increased postruminal starch digestion (Sutton et al., 2000). Harvesting maize at later stages of maturity can have negative impacts on the nutritional quality of the stover. Harika and Sharma (1994) found that delaying the harvest of maize led to a decrease in crude protein content and dry matter degradability, as well as an increase in NDF and ADF content of the leaf and stem fraction of the stover. Similarly, Irlbeck et al. (1993) reported that harvesting maize 28 days after physiological maturity resulted in a higher grain-to-stover ratio; increased stover concentrations of NDF, ADF, and acid detergent lignin (ADL); lower stover yields; and decreased stover concentrations of *in vitro* digestible DM, CP, and total non-structural carbohydrates compared to maize harvested at physiological maturity. Pollmer et al. (1979a) studied the combined ability of flint and dent corn for stover, ear, and grain yield and hypothesized that a high protein percentage and protein yield of mature grain may be due to an intensive N uptake, a prolongation of the N uptake phase, and a high N translocation. Subsequently, Pollmer et al. (1979b) found no significant variation in protein content and early vigor, but they did observe significant variations in other yield components and flowering traits. Flowering time (FTi) in maize is an important and complex agronomical trait critical for crop rotation schemes. Studies have reported that the *ZmMADS1* and *ZmMADS4* genes encoding MADS-box transcriptional regulators are up-regulated in leaves during meristem transition and that their strong over-expression leads to an early-flowering phenotype (Danilevskaya et al., 2008; Alter et al., 2016), especially during long-day conditions. Another reported master regulator of flowering time in maize is *INDETERMINATE1* (ID1), which is a *C2H2* zinc finger protein (Colasanti et al., 1998). The main genetic region that controls the timing of the maize plant transition from vegetative to reproductive growth, known as *Vgt1* (vegetative to generative transition 1), has been identified and cloned. Researchers have found that *Vgt1* carries a specific sequence of DNA called a cis-regulatory element, which controls the expression of *ZmRap2.*7, a gene that inhibits flowering. Interestingly, *ZmRap2.7* is located about 70 kbp downstream from *Vgt1* on the same chromosome. The distribution of different versions (alleles) of *Vgt1* in maize populations is strongly associated with where the plants were originally grown. This suggests that *Vgt1* and associated genetic markers could be useful tools for transferring genes from short-day maize varieties into other types of maize with different flowering times (Jung and Müller, 2009). Harika and Sharma (1994) reported that the number of leaves per plant and the leaf–stem ratio decreased with a delay in harvesting from physiological maturity to the dead ripe stage. Authors have also reported that grain yield showed an increasing trend, whereas cob, stover, total crop residue, and total biomass yield showed a decreasing trend with increasing stages of maturity. The optimum DM content of maize used in ensiling is 300–350 g kg^−1^ (Figure 1). Ensiling maize with a DM <250 g kg^−1^ results in a lower milk yield and protein content. Ensiling >350 g kg^−1^ shows decreased NDF content and increased starch content. The decrease in fiber digestibility could be related to the negative associative effects of higher starch diets on ruminal fiber digestion, which can ultimately lead to ruminal acidosis (Khan et al., 2015).

For silage, plants are harvested before grain ripening. Therefore, it is possible to grow slightly late-maturing genotypes for silage than those grown for grain. A yield improvement in early hybrids can also be accomplished by improving their adaptation to higher plant density (Derieux et al., 1987) coupled with faster rates of leaf production and grain filling.

### Standability

Standability depends upon stalk strength, root lodging, and the leaf area exposed to wind. Maize stalk strength impacts grain yield and silage quality due to its relationship with stalk lodging and stover quality. Dissection of stalk strength into its constituent traits suggests that the structural composition of the rind, and not the pith or total girth, is the chief determinant of strength (Zuber et al., 1980). The mechanical strength of maize stalks depends primarily on the cell wall of the mechanical tissue in the internode rind (Leroux, 2012).

Lodging may occur in stalks and roots. In maize, root lodging occurs more frequently before flowering because of incompletely developed root systems; stem lodging occurs mainly at mid-to-late stages owing to stem senescence and decomposition (Jun et al., 2017). So, for silage maize, root lodging is more important. Root lodging is due to a wind-induced swiveling of the plant base in wet soil conditions, with or without root breaking. Its susceptibility or resistance depends on the quality of the root anchorage, the interactions between root growth or geometry, and the growth or mechanical behavior of the above-ground part of the plant. Resistance also depends on the retardation of root and stalk senescence and on the stiffness of the stalk. Stalk lodging is positively correlated with basal internode length, but negatively correlated with basal internode diameter (Novacek et al., 2013). Koinuma et al. (1998) reported that root lodging control was the main additive effect on lodging resistance. Breeding for resistance to root lodging comprises selection for root number, volume, angle, diameter, and weight (Pellerin et al., 1990). Hebert et al. (2001) also considered that root lodging rate was related to root number, volume, inclination angle, and diameter from stages V12 to R12. Fincher et al. (1985) believed that the determination of vertical root-pulling resistance at stages R1 and R2 could reflect the ability of maize roots to anchor plants. Wang et al. (2022) reported that the root anti-lodging index of maize proved stable from V8 onward during the whole growth period and that vertical leaf area distribution played a substantial role in maize root lodging in terms of wind resultant moment.

The genetic variation regarding resistance to lodging includes large variations in all traits related to the form and structure of the root system, the number of roots on the upper internodes, the average diameter of the primary roots, and the orientation of the growth of the roots in the soil. Zhang et al. (2022) emphasized that lodging resistance is the function of root–shoot interactions and demonstrated that lodging-resistant (LR) and -susceptible (LS) genotypes possessed distinctive morphology and anatomy in the stems and roots. LR maize can allocate more photosynthates to roots and basal stems, which improves stem and root anchorage strength. Brune et al. (2018) proposed that the primary factors for root lodging sensory analysis consisted of root angle, structural rooting depth, soil strength, and wind speed; the secondary factors were plant height, ear height, leaf area, stalk taper, ear mass, and leaf drag; and the tertiary factors were stalk diameter and leaf number. Sposaro et al. (2010) reported that the principal determinants of lodging susceptibility were root plate diameter, stem wall thickness, and the area of the plant loaded by wind gusts.

## Silage quality attributes

For maize to serve as silage, it should fulfill the following criteria for ruminant preference:

• No fungal or mold growth.
• Golden brown color
• Pleasant fruity odor/acceptable aroma. Ammoniacal N levels should not exceed 9%–15% of the total-N as ammonia imparts flavor and aroma to plants.
• Free-flowing and non-sticky texture.
• Mildly acidic taste with an optimum pH of around 4.0–4.5. More lactic acid should be present compared to other acids, and butyric acid levels should be very low, around 0.2%–0.5%.

### Starch composition

Maize grain primarily consists of starch, which makes up around 75% of DMY of the grain, and serves as the primary energy source in the dairy industry. The feeding value of forage maize largely depends on the characteristics of starch degradation (Canizares et al., 2011). The endosperm of corn contains >85% of the starch, which acts as a significant substrate for rumen fermentation, leading to the production of substantial amounts of propionic acid, which is a powerful source of energy (Stevnebo et al., 2006). In whole-plant silage, the typical starch content is around 25%–30% of the total dry matter (NRC, 2001). Increasing starch content and digestibility enhances the performance of dairy cows fed only a corn silage diet (Oba and Allen, 1999a). The starch digestibility is greatly affected by maturity stage, kernel processing, and ensiling period length and is correlated with protein content. Corn stover is usually nutritionally poor over the grain portion of corn. The stover contains limited water-soluble carbohydrates and is low in protein and high in fiber (NDF), with no starch. Starch accumulation increases until the plant reaches physiological maturity. However, when the plant dries down, the starch digestibility decreases. If we skew the ratio of stover to grains peculiar to starch content, maize grain shows a higher contribution toward starch. Therefore, varieties with the right earliness of maturity for their location must be considered along with attributes related to feeding quality. At the same time, we cannot neglect the fact that cell wall digestibility deals with the green parts of the plant where there is a slow release of carbohydrates in the rumen without causing acidosis (due to a high starch content) (Gressley et al., 2011). Dent lines possess more starch degradation than flints due to their floury endosperm and reduced vitreousness (Johnson et al., 1999). One key difference between flint and dent corn is the structure of their endosperm, which is the starchy part of the kernel. Dent corn has a soft, starchy endosperm with a small, indented area on the crown of the kernel. Flint corn, on the other hand, has a hard, vitreous endosperm that is more difficult to be ground into flour. The difference in hard starch in these corn lines makes them differential for digestibility in the rumen of cattle. Philippeau and Michalet-Doreau (1998) compared the ruminal starch degradability of chopped, unensiled, and ensiled grains. Dent corn had a higher digestibility value than flint corn. However, ensiling the corn increased the ruminal starch degradability by an average of 5.8% for both dent and flint corn. Furthermore, the ensiled dent corn showed superior starch digestibility compared to flint corn.

### Crude protein

Protein is the main component of livestock nutrition. As the protein present in silage cannot be measured directly, CP, the amount of nitrogen present in the silage, is used to estimate the amount of true protein and non-protein nitrogen. Maize silage is usually low in protein content (Khan et al., 2015). The bacteria that cause fermentation cannot metabolize silage or fodder if the proper amount of CP is not present. This ultimately affects animal silage intake and reduces the silage digestibility. CP is often used as an indicator of feed quality, but not of energy value. Low CP content can be rectified by supplementing specific oil-seed meals and legume feed.

Season and maturity affect CP concentration. Compared to warmer months, cooler environments will yield fodders with higher levels of CP. Crude protein and fat have larger energy values than carbohydrates (17.57 MJ/kg for carbohydrates, 23.43 MJ/kg for protein, and 39.33 MJ/kg for fats) (NRC, 2001). However, compared to starch or IVNDFD (*in vitro* neutral detergent fiber digestion), the overall contributions of CP of silage maize to the milk yield estimates were lower (Tharangani et al., 2021). Abeysekara et al. (2013) reported a significant genetic variation (62–89 g kg^−1^ DM) in CP content and CP subfractions, mainly soluble protein (425–511 g kg^−1^CP), neutral detergent-insoluble protein (156–220 g kg^−1^ CP), and acid detergent-insoluble protein (50–67 g kg^−1^CP). CP tends to be negatively linked to biomass yield (Figure 1) (Barrière et al., 1997). The genetic variance in CP is particularly small and also shows low narrow-sense heritability. Therefore, the improvement of the CP content of silage maize through conventional genetic tools would likely be very low. An inverse association was observed between crude protein and cell wall components (NDF, hemicellulose) (Saiyad and Kumar, 2018).

### Lignin

Lignin is the polymer of phenylpropanoids, also called monolignols. Guaiacyl (G), syringyl (S), and p-hydroxyphenyl (H) units constitute most of the maize lignin. A distinctive characteristic of grass lignin that affects the cell wall is characterized by the elevated frequency of resistant inter-linkages due to low-quantity H units (Cabane et al., 2004). Lignin is deposited in the cell as the part of cell maturation phase after the elongation phase. It is differentiated from other antinutritional factors as a structural component instead of a secondary metabolite. Lignin provides structural support and strength to the cell wall. It also reduces water loss and entry of disease-causing organisms (Dean and Eriksson, 1994). Due to its negative impact on the nutrient availability of the plant fiber, lignin is regarded as a low-quality component of silage. Lignification regulates the amount of digestible fiber; hence, it has a direct and important impact on the forage’s digestible energy (DE) value (Figure 1) (Jung and Allen, 1995). A fill effect of the diet is observed due to the slow movement of undigested portions of silage. Therefore, lignification reduces the digestive energy concentration in fodder and the amount of dry matter consumed by animals (Moore, 1993).

Lignification is directly or indirectly affected by environmental factors (temperature, soil moisture, light, and soil fertility) and genotype (Nelson and Moser, 1994). According to Wilson et al. (1991), lignification intensifies as more tropical and temperate forage species move into areas with greater temperatures rather than changing the lignin percentages in different tissues.

Brown midrib maize is naturally created by single-gene mutations that impact the lignin biosynthetic pathway (the phenylpropanoid pathway), resulting in its lower lignin content, thereby increasing fiber digestibility. As the name implies, the midrib veins of BMR maize leaves have a unique brown tinge. BMR silage corn has many advantages, such as its favorable impact on the ash, NDF, ADF, and CP levels in maize plants (Weller et al., 1984). BMR maize is also associated with low levels of phenolic acids because of the modification of lignin biosynthesis-related enzymes (Cherney et al., 1991); however, particular hybrids may differ. Disease ratings should be considered while selecting a BMR hybrid as BMR maize is more sensitive to diseases than non-BMR; however, genotypic differences do occur. Oba and Allen, (1999a) stated that among available BMR corn hybrids, homozygous *bm3* was credited for significant improvement in milk production of 4–5 lbs/cow/day. Using the brown midrib lignin mutant (*bm3*), breeding for greater forage digestibility has failed because of the unfavorable correlations between key agronomic variables (Coors et al., 1994). Among commercial BMR corn lines, the Bovalta™ BMR corn hybrid developed by Pioneer has good agronomic characteristics like improved standability and tolerance to foliar diseases. The yield potential of Bovalta™ is good, with an average of 1 ton/acre, which is the highest among commercially available BMR maize today. It also has the lowest dietary undigested fiber (uNDF240) and so was found to increase milk yield (Pioneer, 2022). Other companies like Brevant Seeds and Advanta Seeds also sell BMR forage seeds.

### Fiber and related traits

Fiber is the portion of the cell wall that is partially digested by ruminants (Moore and Hatfield, 1994). The fiber content in silage is measured based on several parameters. Several advancements with better accuracy for phenotyping for silage digestibility traits have been developed. Near-infrared reflectance spectroscopy (NIRS) was more accurate for silage digestibility traits with a high correlation coefficient <0.9 (Zimmer et al., 1990). NIRS was successfully analyzed and calibrated according to the size of the silage particles, which can be used to measure the concentrations of nitrogen, NDF, and *in vitro* fermentability (Montes et al., 2009). Hemicelluloses are solubilized after acid detergent treatment, leaving behind residual acid detergent fiber (ADF) mainly containing cellulose and lignin. A neutral detergent solution is used to digest the residues; the remaining fiber components are lignin, hemicellulose, and cellulose, collectively known as the neutral detergent fiber (NDF) (Van Soest and Wine, 1968). The difference between ADF and NDF content can be used to estimate the hemicellulose content, while the cellulose content can be estimated as the difference between ADF and acid detergent lignin (ADL) after another acid treatment. Alternatively, the whole-lignin content in NDF can be estimated directly as described by Klason lignin (KL) after a single acid treatment (Dence, 1992). *In vitro* NDF digestibility (IVNDFD) is a measure of cell wall digestibility, where a higher value indicates more complete digestion (Mechin et al., 2000). Higher cell-wall lignin and fiber concentrations are negatively correlated with cell-wall digestibility (Wolf et al., 1993; Lundvall et al., 1994). A negative correlation was also observed between crude protein (CP) concentration and fiber components (CF, ADF, NDF, and cellulose) (Jancik et al., 2008). *In vitro* dry matter digestibility (IVDMD) was significantly negatively correlated with fiber components (CF, ADF, and NDF), suggesting that higher lignin concentrations could decrease IVDMD. Increased nitrogen fertilizer application could significantly increase CP and decrease ADF and NDF content (Yolcu and Cetin, 2015; Kaplan et al., 2016; Liu et al., 2018). Cold temperatures could increase ADF and NDF content by causing leaf constriction and cell wall thickening (Kaplan et al., 2016). Breeding for high digestibility in fodder maize could increase animal consumption, growth rate, and milk production (Lundvall et al., 1994). However, a relatively high ADF level could decrease dry matter digestibility, and high NDF content could decrease dry matter intake by animals (Oba and Allen, 1999b; Rotger et al., 2006).

### Micro-nutrients

Micronutrients play important roles in various metabolic processes and help maintain cattle’s fundamental body processes, such as enzyme regulation and chemical biosynthesis. Animal bodies include a wide variety of enzymes that contain zinc or other minerals in the biological systems proteins that depend on zinc to function properly and maintain their structural integrity. Zinc has a role in immunological function, hormone synthesis, cell division, and electrolyte balance in the blood. Similarly, Selenium (Se) is added to animal feed to improve the animals’ nutrition and health.

Research has shown that plant nutrition is an important factor in increasing the Fe and Zn uptake in maize plants, which are then transported to the grain (Cakmak and Kutman, 2018). Increasing the application rate of Fe has little effect on grain Fe, while increasing N application has a positive impact on grain Fe content (Kutman et al., 2010; Erenoglu et al., 2011). N fertilization has also been found to increase the concentration of Zn and Fe in maize shoots and grain, thereby producing silage with higher nutritional value for dairy cattle and reducing the need for micronutrient supplements. However, the inheritance of Fe and Zn concentrations in maize is complex due to environmental and genotype–environment interaction effects. To address this, Drakakaki et al. (2005) demonstrated that the expression of phytase and ferritin genes in transgenic maize can reduce phytate and increase Fe content, respectively.

### Ash content

The ash content of silage is the measure of inorganic non-combustible material such as its total mineral (calcium, phosphorus, potassium, and magnesium) content. Maize silage typically has an ash level of around 5.0%–9.0% of the DM (Figure 1). Although it does not provide calories, the interior ash of plants offers ruminants nutrients like magnesium, calcium, and potassium. Feeding dairy cows with corn silage with high ash content may result in a good uptake of endogenous minerals.

### Fatty acid composition

Most of the energy consumed by ruminants comes from the starch and fiber portions of silage; however, the fat content also has a considerable impact. The fatty acid content and composition of maize silage are highly variable, mainly due to disparities in maturity at harvest (Khan et al., 2012). Various unsaturated fatty acids determine silage quality, with the most pronounced effects determined by α-linolenic, palmitic, and oleic acids. Their optimal concentrations inescapably affect milk quality. Higher concentrations of linolenic acid reduce milk fat through biohydration. Baldin et al. (2018) measured the proportions of fatty acid (FA) concentrations in maize plants, in which 80.5% of the total FA was found in the kernels; 11.8% in the leaves; 5.1% in the stalk; and <2% in the cob, husk, and shank. C_18:2_ (linoleic acid) specifically is contained in the grain (kernel). The selection of corn silages for lower C_18:2_ must focus on decreasing its concentration in the kernel as FA concentration and profile are highly heritable traits in maize. Breeding for commercial silage hybrids with low C_18:2_ is needed; however, at the same time, we must also consider that C_18:2_ is positively correlated with total FA composition (g/kg of DM) in corn silage (Khan et al., 2012). Traditional and genetic modification in plant breeding programs has successfully developed high C_18:1_ and low C_18:2_ in sunflowers. Similarly, maize hybrids with reduced linolenic acid levels did not show reduced starch content and affected the NDF (Alrefai et al., 1995).

### Aroma and color

Good silage emits a slightly sweet or fruity smell due to the presence of lactic acid. Acetic acid is the second most common fermentation end-product that is quite volatile and provides a mild vinegar odor in normal silage. Silage with a rancid, fishy, or putrid odor, yellow-green or dark brown color, and a slimy texture results from clostridial contamination. Clostridia convert lactic acid and excess plant sugars into butyric and acetic acids. Rancid silage typically has reduced energy and protein values. Ethanol is considered one of corn silage’s most significant volatile compounds. Furthermore, alcohols may react with organic acids in the silage, producing esters and adding to the fruity aroma. The limited research indicates a significant correlation between these odors with the levels of ethyl and propyl esters of lactate and acetate and possibly phenylacetic acid (Kung et al., 2018).

### Acidity

Good silage fermentations have a ratio of 2.5–3.0. Lactic acid is the most efficient fermentation acid responsible for reducing the pH of ensiled fresh forage. Moreover, a high level of soluble protein in silage can degrade rapidly and yield ammonia. Ammonia (NH_3_) combines with H+ ions to form ammonium (NH_4_^+^), which helps in preventing the pH of the silage from reaching the desired level (Kung et al., 2018). The ratio of lactic acid to acetic acid is a common indicator of silage quality. Other acids emitted from silage are butanoic, hexanoic, pentanoic, propanoic, and 3-methyl butanoic acids. The higher the total soluble sugar (TSS) in the stalk, the better the aroma and acidity. Increasing sugar content in maize stalk has enhanced the fermentation process with high silage quality. Polymeric carbohydrates of stalk must be broken into simple sugars and converted to lactic acid and ethanol. When the maize stalk has high sugar content (mono, di, and oligosaccharides), the bacteria require less energy to produce lactic acid than complex carbohydrates, leading to efficient fermentation (Bian et al., 2015).

## Approaches for genetic enhancement in silage maize

### Ideotype breeding

Based on the aforementioned desirable characteristics, we can consider a suitable ideotype for silage maize. Silage maize should yield a high and stable amount of digestible organic matter suitable for machine harvest and preservation. Regarding architectural traits, plants need to be designed with certain features; for example, 1) moderate plant height (250–280 cm) and stocky stalk (14–17 leaves/plant), 2) short internodes below the ear and long internodes above the ear; 3) low ear height (55–85 cm); 3) ear-to-plant height ratio ≤ 0.5; 4) pyramided canopy with vertically oriented leaves above the ear and horizontally oriented leaves below the ear; a high leaf: stem ratio, 5) sparse tassel; 6) increased root layers and numbers and vertical roots; 7) rapid rates of cellulose and lignin accumulation; and 8) early cortical formation.

The upright leaf architecture above the ear and the leaves below the ear should be horizontal or show a prostrate leaf orientation for long mid-leaves to have uniform interception of light throughout the plant for enhanced photosynthate efficiency for high biomass yield (Modarres et al., 1998). In silage, the whole plant is used. To increase dry matter yield, it is important to also consider other leaf-related traits, including leaf number, area, and angle, which may affect silage productivity. A smaller tassel with fewer branches is preferred as pollen development uses much energy, which, if saved, leads to increased grain sink (e.g., two ears per plant) to accumulate the surplus. However, if the grain “sink” is limited, this surplus assimilate can be stored in the stover. High contents of non-structural carbohydrates increase the efficiency of utilization. The DM affects the suitability for ensiling. The optimum percentage is 30%–35%, especially from the stover. Breeding for a silage genotype with better ingestibility and cell wall digestibility must involve a comprehensive genetic dissection of cell wall digestibility and friability in its underlying determinants to allow the accumulation of complementary traits (Barrière et al., 2005). Thus, the ideotype should show a limited number of short phytomers, a high leaf: stem ratio, and a slow decrease in the rate of photosynthesis after each individual leaf has fully expanded. A low percentage of water-soluble carbohydrates (5%) is required so that it can be converted into starch in the grains that can limit the losses during preservation (seepage, gaseous losses, and losses after exposure to air) and increase silage digestibility. The ideotype must also be as late-maturing as possible to achieve a large leaf area, prolonged presence of young leaves in the top of the canopy, and delayed emergence of the (light-intercepting) tassel, so as long as ear filling is still assured. The ideotype should also have an early silking date, a large ear, and a slow rate of grain filling (Struik and Deinum, 1982). The ability of plants to stay-green is an indirect way of improving the product output. Root systems must be efficient for water and nutrient uptake and plant standability. Silage hybrids must have an appropriate stalk strength and good resistance to root lodging. Stalk strength is determined by the rind strength and health of the pith tissue. As the pith is generally healthy at silage harvest, the rind strength is primarily responsible for stalk stability. Increased stem diameter is positively correlated with lodging resistance and is negatively correlated with digestibility and dry-matter content. A stocky stem could be considered, with a high pith: rind ratio as, strong pith benefits yield and lodging resistance and also minimizes the adverse effects of thick stems. The amount of the poorly digestible cell wall must be reduced without limiting lodging resistance or the size of the leaf apparatus. Thus, the ideotype should show a limited number of short phytomers, a high leaf: stem ratio, and a slow decrease in the photosynthesis rate after each individual leaf is fully expanded.

### Conventional breeding approaches

Since the beginning of the 19th century, Germany has preserved green fodder, gaining the attention of French agriculturist Auguste Goffart of Sologne (France). The conditions of dairy farming in the United States of America suited the ensiling of green corn fodder; therefore, the first silage was produced in America in 1876 (Crisp and Patterson, 1908). Unlike in these regions, maize was predominantly utilized for human consumption in other parts of the world, leading to a breeding focus primarily on the development of open-pollinated varieties with a strong emphasis on grain yield. Consequently, limited information is available on silage yield, digestibility, and other related traits, and the differentiation of various germplasm groups is unclear. In the Genetic Enhancement of Maize (GEM) project, 35 silage maize hybrids were developed using four elite temperate-adapted lines and checked with five commercial industrial hybrids. Among them, 16 hybrids were highly completive with the industrial hybrids (Perisic et al., 2023). These traits were highly governed by G × E. Perisic et al. (2023) suggested using locally adapted temperate inbreds for developing silage hybrids.

Moreno-Gonzalez et al. (1997) studied the performance and heterosis of flint and early dent germplasm for many traits and reported that dent germplasm exhibited less stalk and root lodging compared to flint germplasm. The breeding efforts for whole-crop silage hybrids in Japan has concentrated on developing new inbred lines by combining the domestic Caribbean flint and Northern flint germplasms with high-yielding capabilities. The selection of genotypes meant for forage should be considered based on genotype × environment interaction (GEI) criteria and their stability/adaptability. With the identification of environments suitable for selection, a breeder can reliably evaluate a large number of genotypes with limited resources (Dhillon et al., 1990a). Forage maize was previously bred primarily for grain yield under the assumption that grain yield is closely correlated with forage yield and quality. However, the breeding strategies for silage and grain use are expected to diverge due to the development and production technologies specific to each type of hybrid (Gurrath et al., 1989; Dhillon et al., 1990b).

Modern hybrids have been shown to possess an average 5.5% lower *in vivo* cell wall digestibility than older hybrids, resulting in an average 2.0% reduction in dry matter digestibility and a tendency to increase grain content. Thomas et al. (2001) conducted a comparative analysis of silage traits between dual-purpose maize and silage maize hybrids. The study found that, except for grain yield, other traits such as high ash content, crude protein, and fiber digestibility showed significant differences. Modern inbred lines with the highest digestibility are expected to serve as the best germplasm for silage hybrids.

Signor et al. (1998) employed the recurrent selection method to cultivate maize silage and expedite the development of different varieties by creating elite populations. From an early synthetic dent, two synthetics were produced: the “base” synthetic with a low selection rate and the “elite” synthetic with a high selection rate. The system achieved two rounds of recurrent selection with a tester, employing multi-trait selection with 1-year assessments of DMY and DMC in three locations. Similarly, Martin and Russel (1984) utilized the recurrent selection method to enhance stalk quality and plant, ear, and grain traits. The authors recommended a mild selection approach for yield and other significant agronomic traits when carrying out a population improvement program for stalk quality.

Genetic improvement is more effective for traits having high heritability. Kapoor and Batra (2015) recorded high heritability along with high genetic advances for plant height, leaf length, leaf width, stem girth, number of leaves, crude protein, acid detergent fiber, dry matter yield, and green fodder yield, including the predominance of additive effects in the inheritance of these traits. Stalk lodging was negatively correlated with flowering dates and stalk-soluble solids, while flowering dates were positively correlated with stalk-soluble solids. This relationship also reflected more heritability; moreover, heritability estimates in comparison with genetic variation values help predict genetic gain under selection (Bekele and Rao, 2014). Therefore, we put forward the fact that the selection of forage genotypes based on stover traits could accordingly be more effective. Lines with favorable alleles will make it possible to reduce the evaluation time. The magnitude of the additive genetic variance will lead to rapid genetic advances during the selection process.

Frey et al. (2004) worked to develop silage maize germplasm with both high whole-plant yield and excellent nutritional quality, including starch content, by selection in the Wisconsin quality synthetic and related maize populations. Theauthors used Wisconsin quality synthetic and related maize populations to improve the stover quality traits including starch. In the first section cycle, the starch content increased from 197 to 214 g/kg. The authors concluded that the Wisconsin population testcrosses were improved by selection for whole-plant and quality attributes, thereby suggesting that it is feasible to develop silage maize germplasm for plant yield and nutritional quality. Dhillon et al. (1990a) reported that flint inbred lines transmitted more favorable digestibility for stover-related traits to the hybrid crosses compared to dent inbred lines. Improving stover digestibility is a crucial objective in the search for genetic variation in maize. The quality of maize is determined by the plant’s morphology and architecture, as the digestibility of plant components varies with genotype. The quality of forage is influenced by various traits, and understanding their heritability is important for developing effective selection procedures. Forage yield is not the only important factor, as the digestibility of improved silage maize varieties is equally crucial. Genetic variation in maize digestibility has been reported, and selecting for important parameters of forage quality and quantity can lead to significant improvements in this trait (Vattikonda and Hunter, 1983; Argillier et al., 1995; Bertoia et al., 2002). Mutants with brown midribs, produced at Purdue University, showed high digestibility and were the first indication of the significant genetic variation in maize digestibility. Gallais et al. (1980) later confirmed this finding in French hybrids and also observed increased intake and feed efficiency in ruminants. The digestibility of stover’s organic matter ranged from 54.9% to 65.2%, and that of the entire crop ranged from 73.3% to 78.3%. The estimated heritability of lignin was 51%–78% in an RIL population (Cardinal et al., 2003). The observed genotypic coefficient of variation (GCV), phenotypic coefficient of variation (PCV), and genetic advancement (GA) were 23.25%, 25.75%, and 47.7%, respectively. A broad-sense heritability of 84.68% was reported by Naharudin et al. (2021). Selecting for high-yielding traits can increase plant lignin content and deteriorate quality due to a significant correlation (0.20–0.36) between biomass yield and lignin content. The high direction of selection resulted in increased levels of crude fiber, cellulose, and lignin. However, measuring fiber and lignin is expensive and time-consuming, and more efficient methods are needed to select for these traits. Production stability should also be considered when breeding forage maize.

Genotypes differ in the protein content of whole plants (Gross, 1980). Schwab et al. (1980) investigated the protein content of different plant parts of some hybrids, and the Illinois High Protein (IHP) strain having 320 g kg^−1^ was obtained after 90 generations of recurrent selection from ordinary maize lines with 80–110 g Kg^−1^ protein (Dudley and Lambert, 1992). Boyat et al. (1980) crossed the IHP strain with French germplasm and followed pedigree selection. As a result, the inbred lines had 20–90 g kg^−1^(16%–21%) higher protein concentration than the check. An early study indicated that low protein percentage showed partial to complete dominance (Frey, 1949). However, other studies reported that additive genetic variance plays a crucial role in protein inheritance (Genter et al., 1957). Recurrent selection for GCA using additive genetic variance will be effective for the improvement of protein content.

### Modern breeding approaches

Genomic selection (GS) is one breeding methodology that helps select superior plants as parents for the next selection cycle using estimated breeding values derived obtained through 1) the parent plant genotypes and 2) the phenotypes and genotypes of their relatives. As a result, GS quickens the breeding process and makes it possible to quickly choose superior genotypes. Thus, GS speeds up the breeding cycle and enables the rapid selection of better genotypes. Contrary to QTL and association mapping, GS uses all the molecular markers for the genomic prediction of the performance of the candidates for selection through predicted breeding and/or genetic values. The major benefits of GS over phenotype-based selection in breeding are reduced cost per cycle and the amount of time needed for variety development (Crossa et al., 2017).

Maize is one of many crop species to profit from GS (Beyene et al., 2015; Vivek et al., 2017). Because of GS implementation, methods for collecting massive amounts of phenotypic data and gene regulatory information have significantly improved (Mejia-Guerra et al., 2012; Tardieu et al., 2017). Taking advantage of new sources of information and adapting the GS approach may be crucial for addressing the rising demand for silage maize on a worldwide scale. Prediction accuracy, determined by the correlation between the breeding value and the genomic estimated values of individuals, varies with different parameters (Zhang et al., 2017). Factors like trait complexity and the degree of similarity between the training and testing sets are important in determining the prediction accuracy. Very few studies on GS have been carried out on maize forage and silage traits. Vinayan et al. (2021) used 276 inbred lines as a training set and developed a prediction model for metabolizable energy and IVOMD. The prediction accuracy was high (r = 0.34–0.42) at different marker densities in the 1024 DH population.

### QTLs and genes identified for silage traits

Several advancements in genome sequencing and statistical analysis have led to the mapping of several major and minor quantitative trait loci. Melchinger et al. (1992) indicated that RFLP-based genetic distance measures can be used to assign inbreds to different heterotrophic groups and seem to be useful for predicting forage yield. Since the later 1990s, several studies have been conducted to detect loci (or QTL) involved in silage traits (Cardinal et al., 2003; Barrière et al., 2007; Riboulet et al., 2008). Schon et al. (1994) found two QTLs and Lubberstedt et al. (1997) found a QTL for crude protein. In a QTL mapping study for yield, earliness, starch, and CP in RILs and top crosses of early dent forage maize, Barrière et al. (2001) identified three QTLs in the top cross and one in the RIL population, which explained 9%–16% of the phenotypic variation (Table 1). No strong colocalization was observed between the QTLs involved in yield and CP. Heterosis effects were proven to be important for CP content in the progenies studied for silage maize. Vinayan et al. (2013) performed a genome-wide association study (GWAS) and reported 10 marker-trait associations (MTAs), with a phenotypic variance ranging from 2.9% to 9.1%. Among the identified regions, the highest percentage of variation in maize CP on chromosome 5 was close to a QTL identified by Xie et al. (2009). Another reported QTL on chromosome 8 for CP content was closer to the region identified in GWAS (Tables 1, 2).

**TABLE 1:** List of QTLs reported in various mapping studies on silage traits in maize.

S.No	Silage traits	Population type	Parents	Chromosome number	No. of QTLs	PVE (%)	References
I	Whole-plant yield						
1	Biomass	F_2∶3_	082/Ye107	1 and 7	5	4.0–15.0	Chen et al. (2011)
2	Fresh stover yield	F_2:3_	8984/GY220 and 8622/GY220	3, 5, 6, 8, and 9	8	6.1–14.9	Wei et al. (2009)
	Dry matter yield	F_2:4_	8984/GY220 and 8622/GY221	2, 3, 7, and 8	6	5.1–11.1	Wei et al. (2009)
3	Relative shoot fresh weight	RILs	Zong3/87-1	1 and 2	3	6.2–11	Liu et al. (2011)
	Relative shoot dry weight	RILs	Zong3/87-1	2 and 9	2	7.2–13.1	Liu et al. (2011)
4	Shoot dry weight	F_2:3_	HZ32/K12	3, 4 and 9	4	3.9–37.3	Qiu et al. (2007)
	Total dry weight	F_2:3_	HZ32/K12	4, 6, and 9	4	5.1–33.3	Qiu et al. (2007)
5	Shoot dry weight	F_2:3_	HZ32/K12	4, 6, 7, and 8	9	5.1–11.7	Osman et al. (2013)
	Total dry weight	F_2:3_	HZ32/K12	2, 4, 5, 6 and 7	9	7.0–11.4	Osman et al. (2013)
6	Shoot fresh weight	RILs	F63/F35	3	1	15.40	Cui et al. (2015)
7	Dry weight	F_2:3_	EP42 (flint)/A661 (dent)	4 and 8	2	6.9–7.1	Rodriguez et al. (2014)
8	Shoot weight	RILs	B73/Mo17	1, 7, 8, and 10	4	14.7	Kaeppler et al. (2000)
9	Straw dry weight	F_2:3_	ETH-DH7/ETH-DL3	1, 2, 5, and 7	5	6.1–14	Leipner et al. (2008)
10	Straw dry weight	F_2_	ETH-DH7/ETH-DL3	3, 5, and 8	4	11.3–15.2	Jompuk et al. (2005)
11	Shoot fresh weight	DH	PH6WC/PH4CV	1, 2, 7, and 9	4	5.4–6.8	Luo et al. (2019)
	Full fresh weight	DH	PH6WC/PH4CV	2, 7, and 9	3	7.0–9.4	Luo et al. (2019)
12	Total biomass	F_2:3_	DTP79/B73	1	1	0.40	Rahman et al. (2011)
13	Shoot dry weight	BC_4_F_3_	Ye478/Wu312	5, 6, 8, 9, and 10	6	5.9–11.9	Cai et al. (2012)
14	Dry matter content	DH and RILs	AS29/AS30, AS07/AS17, and AS06/AS08	1, 2, 3, 4, 5, 6, 7, 8, 9, and 10	40	1.1–18.9	Leng et al. (2018)
	Dry matter yield	DH and RILs	AS29/AS30, AS07/AS17 and AS06/AS08	1, 2, 3, 4, 5, 6, 7, 8, 9, and 10	39	1.8–23.2	Leng et al. (2018)
15	Dry matter yield	F_2_	KW1265/D146	1, 2, 3, 4, 5, 7, 8, 9, and 10	17	3.6–15.5	Lubberstedt et al. (1997)
	Dry matter concentration	F_2_	KW1265/D146	1, 2, 3, 4, 5, 7, 8, and 9	12	4.5–28.7	Lubberstedt et al. (1997)
16	Dry matter yield	RIL	F11/F2	1, 3, 4, and 6	4	12.4–26.7	Riboulet et al. (2008)
				Total	**192**		
II	Plant architecture						
1	Plant height	F_2∶3_	082/Ye107	1, 3, 4, 6, 8, and 9	9	2.0–10.0	Chen et al. (2011)
2	Plant height	F_2:3_	HZ32/K12	1, 2, 4, 6, 7, and 10	8	4.2–13.5	Qiu et al. (2007)
3	Plant height	F_2:3_	HZ32/K12		13	4.1–18.8	Osman et al. (2013)
4	Plant height	F_2:3_	ETH-DH7/ETH-DL3	1, 2, 3, 5, 6, and 8	8	6.2–19.7	Leipner et al. (2008)
5	Plant height	F_2_	ETH-DH7/ETH-DL3	1, 3, 5, 7, and 8	7	7.6–12.7	Jompuk et al. (2005)
6	Plant height	DH and RILs	AS29/AS30, AS07/AS17, and AS06/AS08	1, 2, 3, 4, 5, 6, 7, 8, 9, and 10	52	0.8–23.4	Leng et al. (2018)
7	Plant height	F_2_	KW1265/D146	1, 2, 3, 4, 5, 7, 8, 9, and 10	20	3.6–17.9	Lubberstedt et al. (1997)
				Total	**117**		
III	Leaf traits						
1	Leaf number	RIL	H127 R/Chang7-2	2, 4, 8, 9, 10	5		Li et al. (2021)
2	Total number of leaves	F_2:3_	Huangzao4/HZ32	8, 9	4	0.49–10.11	Xiaobo et al. (2011)
3	Leaf length	F_2:4_	Yu82/Shen137	7, 10	3	8.0–14.3	Ku et al. (2010)
4	Leaf width	F_2:5_	Yu82/Shen137	1, 3, 7, 9	4	8.7–20.4	Ku et al. (2010)
5	Leaf length	BIL	W22/CIMMYT 8759 (*Zea mays* ssp. *parviglumis*)		17	1.2–12.0	Fu et al. (2019)
	Leaf width	BIL	W22/CIMMYT 8759 (*Zea mays* ssp. *parviglumis*)	1, 2, 3, 4, 5, 7, 8, 9, and 10	14	1.2–9.4	Fu et al. (2019)
	Sheath length	BIL	W22/CIMMYT 8759 (*Zea mays* ssp. *parviglumis*)	1, 2, 3, 4, 5, 7, 8, 9, and 10	15	1.1–8.2	Fu et al. (2019)
6	Leaf width	RIL	S-951/Q319	1, 2, 4, 5, 7, 8, and 9	47	6.5–17.0	Liu R et al. (2017)
7	Leaf area	RIL	Xu 178/K12	2, 4, 5, 6, and 7	8	8.8–16.5	Cui et al. (2017)
8	Stay-green	F_6_	IHP1/ILP1	3	1	NA	Zhang et al. (2019)
9	Stay-green	F_2_	Q319/Mo17	1, 2, 3, 5, 8, and 9	14	5.4–11.9	Zheng et al. (2009)
10	Stay-green	RIL	Zheng58/B73	1, 3, 4, 5, 6, 8, and 10	8	4.8–13.5	Yang et al. (2017)
11	Stay-green	RIL and F_2_	CML444/Malawi, CML440/CML504, and CML444/441	1, 3, 4, 5, 8, and 10	8	13–21	Almeida et al. (2014)
				Total	**148**		
IV	Crude protein						
1	Protein content	F_3_	KW1265/D146	1, 2, 3, 5, 6, and 7	8	NA	Schon et al. (1994)
2	Protein content	RIL	F288/F271	1, 2, 4, 6, and 8	5	8.1–18.2	Barrière et al. (2001)
3	Protein yield	F_3_	KW126/D146	1, 3, 4, 6, 7, 8, and 9	7	4.0–23.7	Lu¨bberstedt et al. (1997)
				Total	**20**		
V	Lignin- and fiber-related traits						
1	ADL/NDF	RIL	Zheng 568, HD568	7, 8, 9	5	6.4–11.1	Li et al. (2017)
2	ADL/NDF	RIL	F838/F286	1, 2, 3, 5, 7, 8, and 10	11	5.9–16.5	Barrière et al. (2008)
	KL/NDF	RIL	F838/F286	1, 4, 8, 10	4	5.8–13.2	Barrière et al. (2008)
	pCA	RIL	F838/F286	1, 3, 8, 10	7	5.3–13.3	Barrière et al. (2008)
3	ADL	RIL	F286/F838	1, 2, 3, 4, 5, 7, 8, 9, and 10	34	4.0–21.0	Barrière et al. (2007)
4	IVDOM	F_2_	DO6/D408	2, 5, 6, 8, and 10	6	3.0–18.8	Bohn et al. (2000)
5	NDF	RIL	B73/B52	1, 3, 5, 6, 7, 8, 9, and 10	12	4.4–24.9	Cardinal et al. (2003)
	ADF	RIL	B73/B52	1, 3, 5, 6, 7, 8, 9, and 10	11	4.1–19.0	Cardinal et al. (2003)
	ADL	RIL	B73/B52	1, 2, 3, 6, 7, 8, and 9	16	NA	Cardinal et al. (2003)
6	IVDOM	RIL	F288/F271	3, 4, 6, and 8	5	NA	Fontaine and Briand (2003)
	NDF	RIL	F288/F271	1, 2, 6, and 9	4	NA	Fontaine and Briand (2003)
	KL/ADL	RIL	F288/F271	6, 7, and 9	4	NA	Fontaine and Briand (2003)
	ADL/NDF	RIL	F288/F271	3, 4, 6, and 9	4	NA	Fontaine and Briand (2003)
	pCA	RIL	F288/F271	3, 4, 6, and 9	4	NA	Fontaine and Briand (2003)
	FA	RIL	F288/F271	2, 3, and 4	3	NA	Fontaine and Briand (2003)
7	NDF	F_3_	B73/DE811	1, 3, 4, 7, 8, 9, and 10	11	NA	Krakowsky et al. (2003)
	ADF	F_3_	B73/DE811	1, 3, 4, 7, 8, 9, and 10	12	NA	Krakowsky et al. (2003)
	ADL	F_3_	B73/DE811	1, 3, 4, 7, 8, 9, and 10	10	NA	Krakowsky et al. (2003)
8	NDF	RIL	B73/DE811	1, 2, 3, 4, 5, 6, and 7	16	NA	Krakowsky et al. (2005)
	ADF	RIL	B73/DE811	1, 2, 3, 4, 5, 6, 7, and 10	18	NA	Krakowsky et al. (2005)
	ADL	RIL	B73/DE811	1, 2, 3, 5, 6, 7, and 9	10	NA	Krakowsky et al. (2005)
9	ADL	RIL	B78/De811	1, 2, 3, 4, 5, 7, 9, 8, and 10	14	4.0–16	Krakowsky et al. (2006)
	NDF	RIL	B78/De811	1, 2, 3, 4, 6, 9, and 10	12	4.0–17.0	Krakowsky et al. (2006)
	ADF	RIL	B78/De811	1, 3, 4, 5, 6, 8, 9, and 10	13	5.0–12.0	Krakowsky et al. (2006)
10	ADL/NDF	RIL	F11/F2	1, 2, and 4	4	10.7–19.7	Riboulet et al. (2008)
	IVNDFD	RIL	F11/F2	2 and 6	2	7.7 and 9.4	Riboulet et al. (2008)
	DINAGZ	RIL	F11/F2	2 and 6	2	14.5 and 17.5	Riboulet et al. (2008)
11	NDF	RIL	F288/F271	1, 3, and 9	3	8.9–16.5	Roussel et al. (2002)
	Hcell/NDF	RIL	F288/F271	2, 4, 6, and 9	4	11.3–27.7	Roussel et al. (2002)
	Cell/NDF	RIL	F288/F271	1, 2, 4, 6, and 9	6	7.8–17.5	Roussel et al. (2002)
	ADL/NDF	RIL	F288/F271	3, 6, and 9	4	7.2–20.4	Roussel et al. (2002)
	KL/NDF	RIL	F288/F271	1, 6, and 9	3	11.8–26.1	Roussel et al. (2002)
	IVDMD	RIL	F288/F271	1, 3, 6, and 9	5	7.5–31.7	Roussel et al. (2002)
	DINAGZ	RIL	F288/F271	1, 2, 3, 4, 6, and 9	7	6.6–26.0	Roussel et al. (2002)
	IVNDFD	RIL	F288/F271	1, 3, 6, and 9	4	6.6–40.2	Roussel et al. (2002)
12	ADF	RIL	B73/By804	2, 6, 7, and 8	4	6.3–12.2	Wang et al. (2020)
	ADL/NDF	RIL	B73/By804	1, 2, 6, and 7	5	1.5–20.0	Wang et al. (2020)
	CEL/NDF	RIL	B73/By804	1 and 6	3	6.4–12.4	Wang et al. (2020)
	IVDMD	RIL	B73/By804	2 and 6	2	9.3 and 13.0	Wang et al. (2020)
	IVNDFD	RIL	B73/By804	10	1	10	Wang et al. (2020)
	NDF	RIL	B73/By804	2, 6, 7, and 8	5	6.0–13.3	Wang et al. (2020)
				Total	**310**		
**VI**	Starch digestibility						
1	Starch degradability traits	DH	AS06 (Flint)/AS08 (Dent)	1, 2, 3, 4, 5, 7, 8, 9, and 10	101	NA	Leng et al. (2019)
2	Starch and soluble carbohydrate content	RIL	F288/F271	1, 2, 3, 5, 6, and 7	15	6.6–15.2	Barrière et al. (2001)
3	Starch concentration	F_2_	KW1265/D146	1, 2, 3, 7, 8, 9, and 10	15	4.5–16.9	Lu¨bberstedt et al. (1997)
4	Starch yield	F_2_	KW1265/D146	1, 4, 5, 7, 8, 9, and 10	12	3.1–12.4	Lu¨bberstedt et al. (1997)
				Total	**143**		

DH, double haploid; RIL, recombinant inbred lines; ADL, acid detergent lignin; ADF, acid detergent fiber; NDF, neutral detergent fiber; KL, Klason lignin, pCA, p-coumaric acid; FA, ferulic acid; IVDOM, *in vitro* digestibility of organic matter; IVNDFD, *in vitro* NDF digestibility; IVNDFD, *in vitro* NDF digestibility; cell, cellulose; DINAGZ, *in vitro* digestibility of non-parts (starch, soluble carbohydrate, and crude protein) and IVDMD, *in vitro* dry matter digestibility.

Bold values in table indicate the sum total of QTLs reported for respective mentioned trait(s).

**TABLE 2:** Genome-wide association studies on maize silage traits.

S.No	Association panel name	Population size	Traits	SNPs used	Marker-trait associations (MTAs) identified	References
1	DTMA panel	276	CP	443 k	10	Vinayan et al. (2013)
	DTMA panel	276	ADF	443 k	10	Vinayan et al. (2013)
	DTMA panel	276	IVOMD	443 k	10	Vinayan et al. (2013)
2	AM368	368	LIG	56 k	22	Li X. et al. (2016)
	AM368	368	CEL	56 k	18	Li X. et al. (2016)
	AM368	368	HC	56 k	24	Li X. et al. (2016)
3	IAP	276	pCA	246 k	5	López-Malvar et al. (2019)
	IAP	276	FA	246 k	7	López-Malvar et al. (2019)
	IAP	276	DFA	246k	12	López-Malvar et al. (2019)
	IAP	276	DFAT	246 k	2	López-Malvar et al. (2019)
4	MAGIC	408	Stover yield	215 k	13	López-Malvar et al. (2021)
5	IAP	276	IVOMD	181 k	7	Vinayan et al. (2021)
6	IAP	453	PH	899 k	6	Mazaheri et al. (2019)
	IAP	453	Stalk diameter	899 k	9	Mazaheri et al. (2019)
7	—	400	Leaf senescence	1000 k	10	Yannam et al. (2022)

DTMA, stress-tolerant maize for Africa; AM, association mapping; IAP, inbred association panel; MAGIC, multi-parent advanced generation intercross; CP, crude protein; IVOMD, *in vitro* digestibility of organic matter; LIG, lignin; CEL, cellulose; HC, hemi- cellulose; pCA, p-coumaric acid; FA, ferulic acid; DFA, ferulic acid dimers; DFAT, total ferulic acid dimers; PH, plant height.

Cell wall architecture plays a key role in forage digestibility. Lignin, cellulose, and hemicellulose are the three main components of plant cell walls and can impact stalk quality by affecting cell wall structure and strength. However, cell wall formation is a dynamic process and occurs throughout the growing period. Thus, the genetic analysis of the cell wall and digestibility traits at some specific stages might not provide a reasonable explanation for forage quality. Among cell wall components, cell wall-bound hydroxycinnamates derived from the phenylpropanoid pathway play crucial roles in reinforcing the structural integrity of the cell wall. Additionally, these wall-bound hydroxycinnamates not only contribute significantly to defense against pests and pathogens but also impact forage digestibility. More than 300 QTLs were reported for various fiber traits (ADF, NDF, and ADL) which explained variance in phenotype ranging from 3.0% to 40.2% (Table 1). In their meta-analysis of QTLs associated with maize silage quality traits, Truntzler et al. (2010) identified numerous meta-QTLs (MQTLs) for digestibility and cell wall composition traits (26 and 42 MQTLs, respectively). These MQTLs were distributed across 10 chromosomes, co-localized with carbohydrate metabolism genes (*Zmmur3, ZmXTH*, glucosidases, UDP decarboxylases, and a-L-arabinofuranosidase) and lignin synthesis (*ZmCAD2, ZmCCoAOMT2, ZmCOMT, ZmC3H*, and *ZmF5H*) responsible for cell wall components and digestibility. This study emphasized the genetic complexity of silage quality traits, which mainly involve QTL with small effects.

**FIGURE 2:**
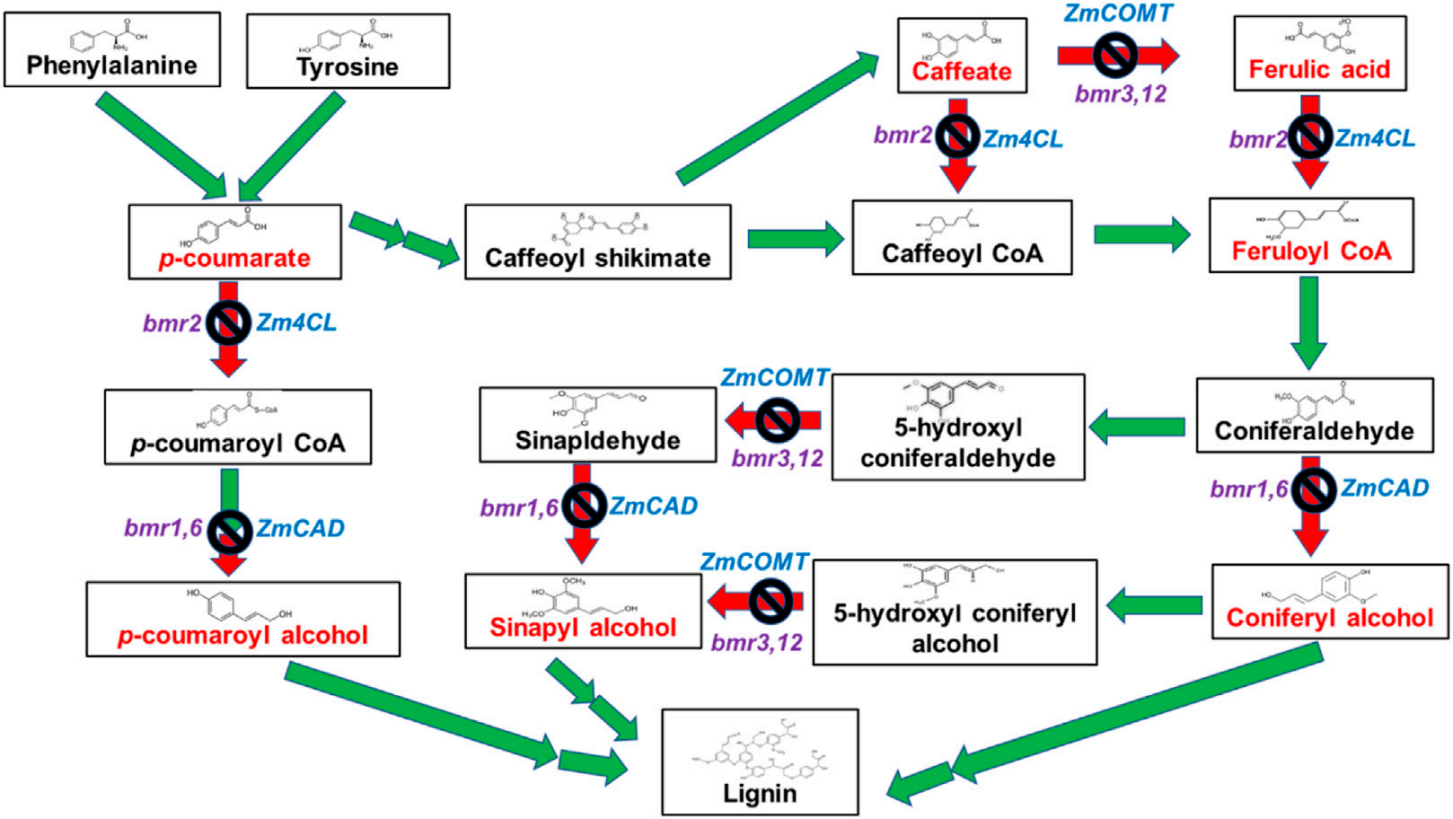
Genes in the lignin biosynthetic pathway targeted for creating different bmr mutants. The red arrow with a block symbol indicates the knockout of genes in the lignin biosynthesis pathway.

**FIGURE 3:**
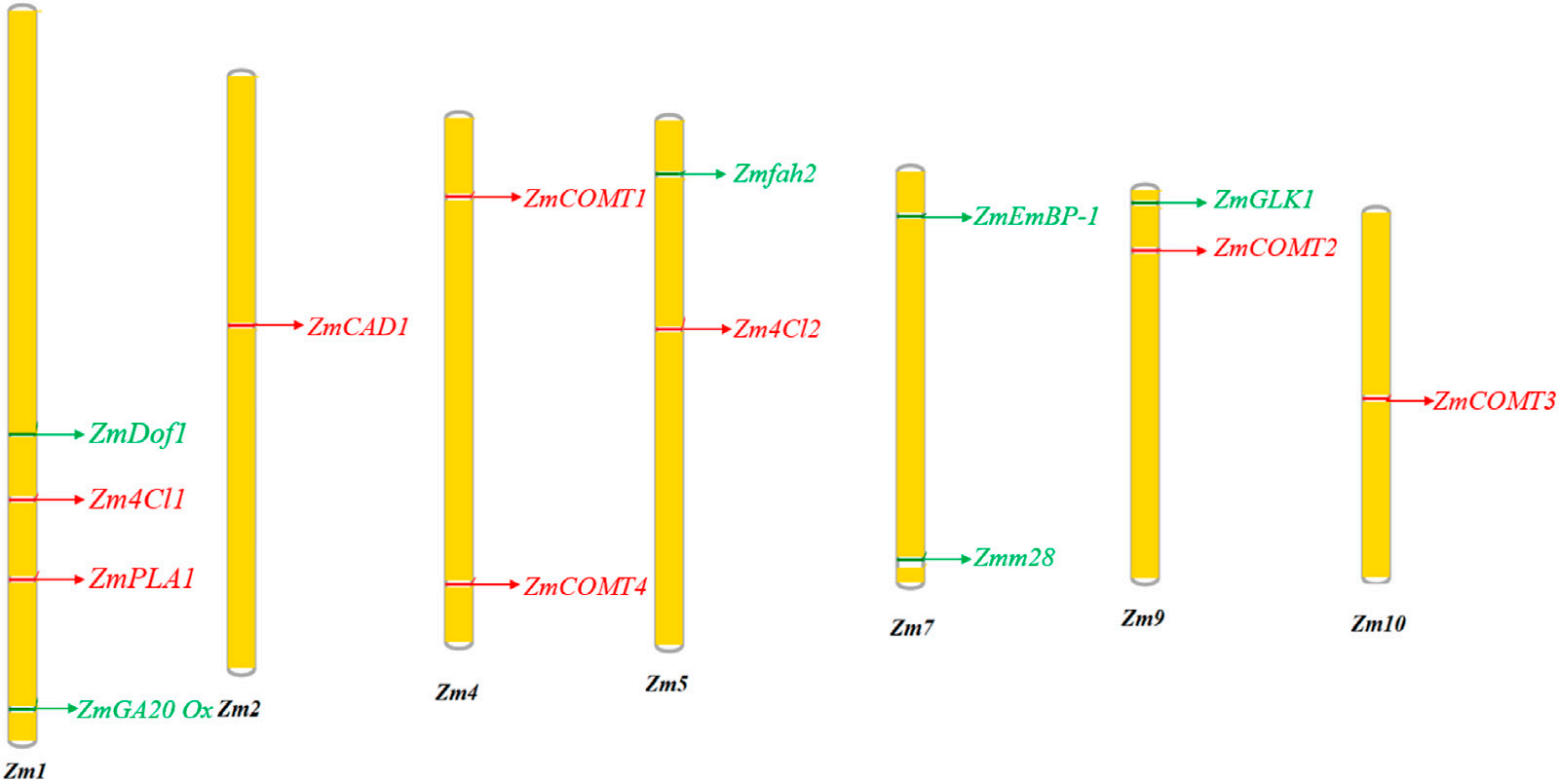
Location of genes targeting bmr and the accumulation of increased biomass in maize chromosome. The green regions in the chromosomes are genes for increasing the biomass. The red regions are genes in the lignin biosynthetic pathway.

The phenylpropanoid pathway involved in the lignin synthesis of maize has been studied in detail. Many enzymes are required for monolignol biosynthesis through the phenylpropanoid pathway, including phenylalanine ammonia-lyase; cinnamate 4- hydroxylase (C4H); 4-coumarate-CoA ligase (4CL); cinnamoyl CoA reductase (CCR); hydroxycinnamoyl CoA: shikimate hydroxycinnamoyl transferase (HCT); coumarate 3-hydroxylase (C3H); caffeoyl CoA 3-O-methyl transferase (CCoAOMT); ferulate 5-hydroxylase (F5H); caffeic acid 3-O-methyltransferase (COMT); and cinnamyl alcohol dehydrogenase (CAD). Brown midrib genotypes contain mutations in the genes coding for the aforementioned enzymes. In the case of bmr1, the expression of the cinnamyl alcohol dehydrogenase (CAD) gene (Halpin et al., 1998) was highly affected (Halpin et al., 1994). Downregulating this gene allows the creation of a maize silage genotype with modified lignin and high digestibility (Table 3). The different BMR mutants obtained by targeting genes are briefly explained in Figures 2, 3 **.**

**TABLE 3:** List of genes targeted by biotechnological approaches to create bmr maize lines with high biomass.

S.No	Genes	Gene names	Gene ID (chromosome position)	Physical position	Mechanism	Role in maize	References
1	Digestibility						
1	*Zmfah2*	Ferulic acid 5-hydroxylase2	Zm00001eb220390 (5)	23,623,860–23,627,256	Gene knockout	Lignin biosynthesis	Christensen and Rasmussen (2019)
2	*ZmCAD1*	Cinnamyl alcohol dehydrogenase1	Zm00001eb071040 (2)	10,925,155–10,929,339	Gene knockout	Lignin biosynthesis	Christensen and Rasmussen (2019)
3	*ZmCOMT1*	Caffeoyl CoA 3-O-methyl transferase 1	Zm00001eb172420 (4)	34,198,723–34,201,171	Gene knockout	Lignin biosynthesis	Christensen and Rasmussen (2019)
4	*ZmCOMT2*	Caffeoyl CoA 3-O-methyl transferase 2	Zm00001eb375610 (9)	17,958,934–17,960,272	Gene knockout	Lignin biosynthesis	Christensen and Rasmussen (2019)
5	*ZmCOMT3*	Caffeoyl CoA 3-O-methyl transferase3	Zm00001eb416160 (10)	81,688,871–81,699,884	Gene knockout	Lignin biosynthesis	Christensen and Rasmussen (2019)
6	*ZmCOMT4*	Caffeoyl CoA 3-O-methyl transferase4	Zm00001eb199470 (4)	204,194,919–204,196,509	Gene knockout	Lignin biosynthesis	Christensen and Rasmussen (2019)
7	*Zm4Cl1*	4-Coumaric acid: coenzyme A ligase 1	Zm00001eb040790 (1)	214,915,554–214,917,940	Gene knockout	Lignin biosynthesis	Christensen and Rasmussen (2019)
8	*Zm4Cl2*	4-Coumaric acid: coenzyme A ligase 2	Zm00001eb233720 (5)	91,220,184–91,225,235	Gene knockout	Lignin biosynthesis	Christensen and Rasmussen (2019)
2	Whole plant yield						
1	*ZmPLA1*	Cytochrome P-450	Zm00001eb048540 (1)	249,356,525–249,358,967	Over-expression	Biomass accumulation	Sun et al. (2017)
2	*ZmDof1*	DNA-binding with one finger1	Zm00001eb033670 (1)	186,920,136–186,921,569	Over-expression	Biomass accumulation	Pena et al. (2017)
3	*ZmGLK1*	GOLDEN2-like transcription factor	Zm00001eb371980 (9)	4,107,676–4,111,299	Over-expression	Biomass accumulation	Li et al. (2020)
4	*ZmEmBP-1*	EM-binding protein1	Zm00001eb303220 (7)	19,797,398–19,802,247	Over-expression	Biomass accumulation	Perveen et al. (2020)
5	*Zmm28*	MADS-transcription factor 67	Zm00001eb327040 (7)	173,406,644–173,412,313	Over-expression	Biomass accumulation	Wu et al. (2019)
6	*ZmGA20* Ox	GA 20 Oxidase	Zm00001eb064970 (1)	306,529,697–306,531,340	Over-expression	Biomass accumulation	de Freitas Lima et al. (2017)

GWAS played an important role in dissecting complex quantitative and qualitative traits in plants due to its faster analyses, numerous high-resolution markers, and abundant genomic and phenotypic variation. Various candidate genes have been identified by genome-wide studies for cell wall digestibility and degradability, and ADF, NDF, and IVDMD of the stalk have been evaluated in a diverse maize population. The C3H gene (*ZmC3H2*) was found to be directly involved in cell wall component biosynthesis. *ZmPox3* and KAPP, as candidate genes, were also found to be involved in signal transduction, stress resistance, and transcriptional regulation of the expression of genes responsible for cell wall biosynthesis (Wang et al., 2016). Li X. et al. (2016) identified around 22, 18, and 24 MTAs as significantly associated with lignin, cellulose, and hemicellulose, respectively. Moreover, the candidate genes identified were known to encode enzymes that are mainly involved in the metabolism of the cell wall, various transcription factors, protein kinase enzyme, and proteins related to various biological pathways in maize (Li X. et al., 2016; Karnatam et al., 2020). López-Malvar et al. (2019) reported 26 MTAs for the components of lignin biosynthesis. López-Malvar et al. (2019) identified SNPs significantly associated with low stem hydroxycinnamate, which explained a low percentage of total phenotypic variability (7%–10%) but indirect selection based on cell wall kinases (WAKs) and members of the receptor-like cytoplasmic kinase (RLCK), acting as cell wall modulators, proved to be potential candidate genes for cell wall-bound hydroxycinnamate accumulation (Lopez-Malvar et al., 2019) (Table 2).

Leng et al. (2019) mapped seven (seedling fraction) to nine (vitreousness) QTLs for starch degradability traits in a DH population. These QTLs were distributed across all 10 chromosomes, except for chromosome 6, and explained phenotypic variance between 38.2% and 76.5%. Studies have shown that the starch from genotypes having *su2* and *wx* genes can be quickly degraded and that degradability increases five-fold, when combining both genes (Inouchi et al., 1987). Wong et al. (2004) reported QTLs explaining micronutrient variation in maize populations. The major QTLs were reported on chromosomes 6 and 7.

Regarding improving quantitative traits, several QTLs for biomass accumulation have been reported under normal and stress conditions such as waterlogging and salinity (Chen et al., 2011; Luo et al., 2019). Sixteen QTL mapping studies reported 188 QTLs for biomass and related traits such as dry matter (Table 1). Moreover, 117 QTLs for plant height were reported in seven studies with explained phenotypic variances ranging from 0.8% to 23.4%. Similarly, 117 QTLs for leaf length, width, and area and sheath length were reported in seven studies. Cui et al. (2017) identified two important QTLs associated with leaf number above the ear (LAE), *qLA3-4* and *qLA7-1*, harboring the dominant gene *lfy1* on chromosome 3, which is responsible for additional leaves above the ear in leafy maize. Four LAE QTLs were discovered to overlap with plant height and/or flowering time, which suggested the potential pleiotropic effects of these QTLs. These findings improve our comprehension of the trait genetic architecture influencing maize LAE and the creation of hybrids with greater LAE to increase the photosynthetically active surface and boost photosynthate accumulation. Among the seven studies on leaf area, some QTLs such as *Yabby15, ZmPIN1b, Ig1, mrl1, drl 1*, and *drl 2* co-localized with characterized genes for leaf traits*.* As candidate genes for biomass and leaf-related QTLs have not been studied in detail, meta-QTL analysis can provide genomic regions governing biomass accumulation. These regions can be targeted for candidate gene mining, and meta-QTL regions can also be used as fixed effects in GS (Karnatam et al., 2023).

Mazaheri et al. (2019) reported nine and six MTAs for maize stalk diameter and plant height, respectively. In the GWAS study, the MTAs contained genes like *Zmm22* and *Fpa* associated with plant height and stalk diameter. *Zmm22* expression is reported to modulate reproductive transition in maize (Kaeppler et al., 2020). The identified candidate genes were found to be associated with fatty acid hydroxylase activity and the MAPK signaling pathways (Table 2).

Modulating the expression of the maize PLASTOCHRON1 (*ZmPLA1*) gene, which encodes cytochrome P450 (CYP78A1), results in increased plant organ growth, seedling vigor, stover biomass, and seed yield. The underexpression of *GA 20-oxidase* involved in gibberellic acid degradation increased the total biomass accumulation. The silencing of maize *GA 20-Oxidase* expression promoted faster growth compared to its overexpression (de Freitas Lima et al., 2017). Maize *Dof1* (*ZmDof1*) has been shown to upregulate the expression of phosphoenolpyruvate carboxylase (PEPC), the initial carbon-fixing enzyme of C4 species and a key component of the TCA cycle. In the case of transgenic wheat and sorghum, *ZmDof1* over-expression showed increased fresh leaf and dry biomass accumulation by increasing the photosynthetic efficiency (Pena et al., 2017). Similarly, *ZmGLK1* improved photosynthetic capacity, enhanced carbohydrate accumulation, and increased biomass and grain yield. Transgenic rice plants showed increased carbohydrate levels and a 30%–40% increase in both vegetative biomass and grain yield (Li et al., 2020). López-Malvar et al. (2021) reported 13 MTAs associated with stover yield distributed across all chromosomes. Genes of transcriptional factors such as *ZmEmBP-1*and *Zmm28* showed high photosynthetic rates through the overexpression of photosynthetic genes and CO_2_ assimilation (Perveen et al., 2020). These QTL and putative genes may offer useful data for MAS and GS to improve plant architecture and composition to enhance silage production (Table 3).

### Biotechnological approaches

Maize is one of the most important and extensively studied cereals, known for being one of the most tractable genetic systems among monocots. The development of biotechnology has led to a great increase in our knowledge of maize genetics and understanding of the structure and behavior of maize genomes. The maize whole-genome sequence assembly was successfully completed in 2009 (Schnable et al., 2009). The latest version of maize whole-genome assembly, Zm-B73-REFERENCE-NAM-5.0, was released in 2020. As a whole-genome sequencing application, maize DNA markers have proven to be tools for various analyses ranging from phylogenetic analysis to positional cloning of genes. In biotechnology applications, maize markers have been used to characterize germplasm, perform DNA fingerprinting, map quantitative trait loci, perform MAS, identify candidate genes, etc. (Drinc et al., 2004). In the North and South American continents, transgenic maize is commercially cultivated and has several stacked transgenes for insect resistance (corn root worm and Lepidopteran) and several herbicide resistances (glyphosate, glufosinate, and sulfonylurea). These transgenic maize hybrids are commercially available as Agrisure^Tm^, Herculex^Tm^, Yield gard^Tm^, and Bt Xtra^Tm^ (ISAAA, 2022). Apart from transgenic technology, several genome editing success stories have been reported for early flowering (McCaw et al., 2021), herbicide resistance (Li et al., 2020), aromatic maize (Wang et al., 2021), starch chemistry (Qi et al., 2020), and disease resistance. The private seed company, Corteva Ltd., has developed waxy corn hybrids through CRISPR/cas9 knockout of genes responsible for high amylopectin synthesis (Grain, 2021).

**FIGURE 4:**
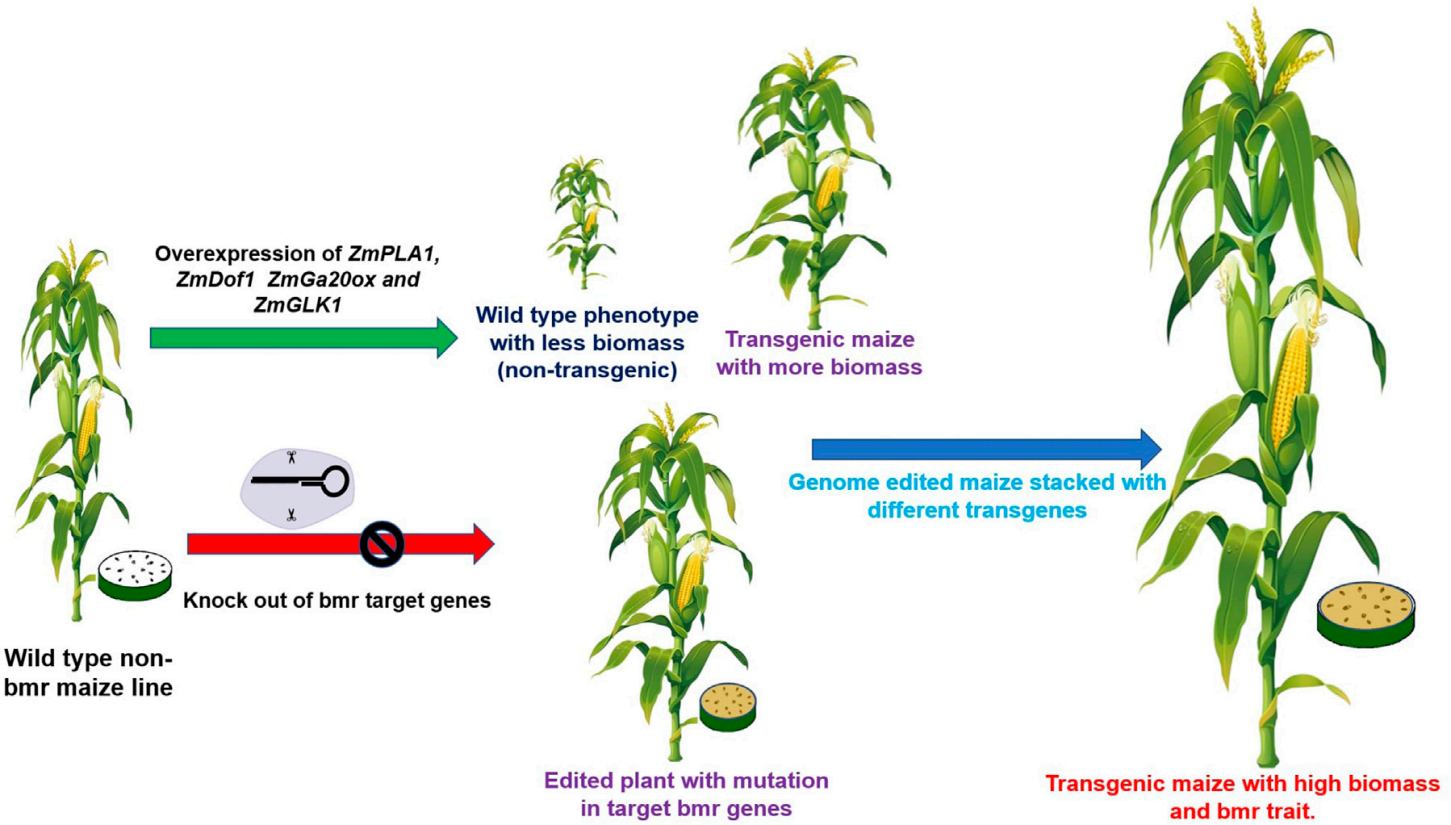
Biotechnological approaches for the development of maize with high-quality silage.

The development of high digestibility in forage maize is important because it improves animal intake, growth rate, and milk production (Lundvall et al., 1994). Several genetic approaches are available for enhancing the digestibility of forage maize, including 1) using known mutants of the lignin pathway; 2) manipulating genes in the lignin, cellulose, and hemicellulose pathways via genetic engineering; and 3) breeding for lower fiber and lignin concentrations with conventional methods or MAS. For example, breeding for higher forage digestibility using the brown midrib lignin mutant (*bm3*) has proven unsuccessful due to the undesirable correlated effects of these genotypes on important agronomic traits (Coors et al., 1994). Hence, modern biotechnological tools such as CRISPR/Cas 9, RNAi, TALENs, and ZFNs can be used for site-directed mutagenesis to either knock out genes or perform base editing (Figures 2, 4) (Premnath et al., 2022).

Using genetic engineering, plants with overexpression of genes such as *ZmPLA1, ZmDof1, ZmGLK1, ZmEmBP-1,* and *Zmm28* showed higher biomass accumulation. In addition, downregulating some genes such as *GA 20 oxidase* and some genes in lignin synthesis can improve the quality and quantity of silage. Using biotechnological tools like genetic engineering and genome editing, several genes can be targeted to develop the best maize lines for silage making, as illustrated in Figures 2, 4. To develop BMR mutants, different genes involved in the phenylpropanoid pathway can be knocked out by CRISPR/Cas9 technology (Table 3).

## Conclusion

The proposed ideotype and described quality parameters in this study can be used as a useful resource for breeding silage maize. Furthermore, the QTL and candidate genes mentioned in this work can be targeted for genetic enhancement. BMR maize is known for its digestibility, which is generally identified in natural populations or using mutagens to create mutations in the genes. The phenylpropanoid pathway of maize is well-studied; therefore, the desired *BMR* genes can be targeted and editing can be carried out in the parents of hybrids. American and European countries dominate the corn silage market because of their diversity and robust germplasm for silage. The demand for silage in Asian countries has been steadily increasing in recent years, but the progress of breeding silage maize is slow due to several factors. Institutes in Asian countries could collaborate with private seed sectors to effectively utilize maize germplasm to breed silage corn. The utilization of temperate germplasm to increase maturity duration and biomass is a viable option. The feasibility of sharing maize silage genetic stocks of American and European origin for Asian silage breeding programs may result in the rapid delivery of improved products to farmers.

Silage plays a critical role in livestock nutrition as it serves as a preserved source of easily digestible nutrients in diets for high-producing livestock, ensuring optimal rumen function. Additionally, it serves as a supplementary feed during winter and drought periods. With the rising global demand for silage driven by the growing world population, the increasing size of livestock farms, and the need to secure global food security, it is essential to foster multidisciplinary collaborations between experts in plant breeding, mechanical engineering, chemistry, biochemistry, microbiology, and animal nutrition to develop innovative solutions to improve the efficiency, quality, and safety of silage production, ultimately benefiting farmers, consumers, and industry. Numerous studies have revealed that grain yield alone is not the most accurate indicator of a hybrid’s performance for silage. While significant strides have been made in improving grain yield over the past 50 years, further research is needed to develop hybrids specifically for silage production. Future prospects include the creation of high-output silage hybrids, enhancing food safety and animal health by improving the hygienic quality of silage, and mitigating environmental risks.
